# The Ergogenic Effects of Transcranial Direct Current Stimulation on Exercise Performance

**DOI:** 10.3389/fphys.2017.00090

**Published:** 2017-02-14

**Authors:** Luca Angius, James Hopker, Alexis R. Mauger

**Affiliations:** Endurance Research Group, School of Sport and Exercise Sciences, University of KentChatham, UK

**Keywords:** tDCS, brain stimulation, exercise performance, perception of effort, cortical excitability, motor cortex

## Abstract

The physical limits of the human performance have been the object of study for a considerable time. Most of the research has focused on the locomotor muscles, lungs, and heart. As a consequence, much of the contemporary literature has ignored the importance of the brain in the regulation of exercise performance. With the introduction and development of new non-invasive devices, the knowledge regarding the behavior of the central nervous system during exercise has advanced. A first step has been provided from studies involving neuroimaging techniques where the role of specific brain areas have been identified during isolated muscle or whole-body exercise. Furthermore, a new interesting approach has been provided by studies involving non-invasive techniques to manipulate specific brain areas. These techniques most commonly involve the use of an electrical or magnetic field crossing the brain. In this regard, there has been emerging literature demonstrating the possibility to influence exercise outcomes in healthy people following stimulation of specific brain areas. Specifically, transcranial direct current stimulation (tDCS) has been recently used prior to exercise in order to improve exercise performance under a wide range of exercise types. In this review article, we discuss the evidence provided from experimental studies involving tDCS. The aim of this review is to provide a critical analysis of the experimental studies investigating the application of tDCS prior to exercise and how it influences brain function and performance. Finally, we provide a critical opinion of the usage of tDCS for exercise enhancement. This will consequently progress the current knowledge base regarding the effect of tDCS on exercise and provides both a methodological and theoretical foundation on which future research can be based.

## Introduction

During sustained submaximal contraction, the excitability of spinal motoneurons and the contractile capacity of the muscle fibers are reduced (Butler et al., 2003; Allen et al., 2008), so that in order to maintain the required force or power, the input to the spinal motoneurons must increase (Taylor et al., 1996). This input (also called descending drive) is likely to originate from the corticospinal pathway, and previous experiments have demonstrated a number of factors which may moderate this (Gandevia, 2001; Enoka et al., 2011). In this regard, a failure to generate output from the motor cortex (M1) has been defined as supraspinal fatigue, and together with peripheral mechanisms, participates in muscle fatigue (Gandevia, 2001). Previous studies have suggested that the development supraspinal fatigue is accompanied by changes in motor cortex excitability (Taylor et al., 1996).

Interventions that increase M1 excitability might increase the output from M1 (increase descending drive) thus delaying the development of supraspinal fatigue and therefore improving exercise capacity (Cogiamanian et al., 2007; Williams et al., 2013). In this regard, a neuromodulatory technique called transcranial direct current stimulation (tDCS) has been widely used to modulate the excitability of a targeted brain area through the application of a weak electrical current across the scalp. The electrical current alters the resting membrane potential of the targeted neurons, with the anodal electrode being excitatory and the cathodal being inhibitory (Nitsche et al., 2008; George and Aston-Jones, 2010). These effects can persist for up to 90 min following 9–13 min of stimulation (Nitsche and Paulus, 2001). Studies have demonstrated that acute tDCS is a safe neuromodulatory brain technique, with no or only minor side effects (Fregni et al., 2006; Poreisz et al., 2007; Palm et al., 2008; Frank et al., 2010) and is both cheap and easy to administer. Therefore, interest in tDCS' ergogenic potential has grown considerably.

Research has only recently started to investigate the effect of tDCS on physical performance and, given the prominent role of the motor and premotor brain regions in the development of supraspinal fatigue (Gandevia, 2001), most studies have attempted to target these areas. To date, there are a limited number of studies, showing inconsistent results and often with flawed methodological design. Nevertheless, the balance of evidence suggests that tDCS might have a positive effect on exercise capacity. A summary of the most significant studies on tDCS stimulation and exercise performance are shown in Table 1. For the purpose of this review we considered studies that adhered to the following criteria:

- Acute administration of tDCS prior to, or during, exercise in healthy participants;
- Continuous exercise lasting at least 75 s (Gastin, 2001);
- Exercise tasks involving time to exhaustion, time trial, or incremental exercise testing.

**Table 1:** **List of tDCS studies on exercise performance**.

**Articles**	**Sample size**	**Placement of electrodes**	**Stimulation duration**	**Stimulation intensity (mA)**	**Electrode size (cm^2^)**	**Control condition**	**Muscle group investigated**	**Exercise protocol**	**Performance result**
Cogiamanian et al., 2007	Study 1, *n* = 9; study 2, *n* = 15	Anodal right M1, cathodal right shoulder	10 min	1.5	35	Cathodal and control	Left elbow flexors	Isometric TTF at 35% MVC	Improvement
Muthalib et al., 2013	*n* = 15	Anodal right M1, cathodal right shoulder	10 min	2	24	Sham	Left elbow flexors at 90° flexion	Isometric TTF at 30% MVC	No improvement
Kan et al., 2013	*n* = 15	Anodal right M1, cathodal contralateral shoulder	10 min	2	24	Sham	Elbow flexors at 90° flexion	Isometric TTF at 30% MVC	No improvement
Williams et al., 2013	*n* = 18	Anodal right M1, cathodal left forehead	20 min during TTF	1.5	35	Sham	Left elbow flexors	Isometric TTF at 20% MVC	Improvement
Okano et al., 2015	*n* = 10	Anodal T3, cathodal over Fp2	20 min	2	35	Sham	Lower limbs	Cycling, from 15W +25 Wmin^−−1^	Improvement of ~4%
Angius et al., 2015a	*n* = 9	Anodal right M1, cathodal Fp2	10 min	2	35	Sham and control	Lower limbs	Cycling, at 70% of peak power	No improvement
Vitor-Costa et al., 2015	*n* = 11	Active over Cz and reference over occipital protuberance	13 min	2.0	35	Sham and cathodal	Lower limbs	Cycling, at 80% peak power	Improvement
Abdelmoula et al., 2016	*n* = 11	Anodal left M1, cathodal right shoulder	10 min	1.5	35	Sham	Elbow flexors	Isometric TTF at 35% MVC	Improvement
Oki et al., 2016	*n* = 13	Anode over right M1, cathode over the left forehead	Max 20 min during TTF	1.5	35	Sham	Elbow flexors	Isometric TTF at 20% MVC	Improvement
Angius et al., 2016a	*n* = 12	Bilateral montage, active electrode over M1 and reference over the ipsilateral shoulder	10 min	2.0	35	Sham and cathodal	Lower limbs	Cycling, at 70% of peak power	Improvement
Barwood et al., 2016	Study 1, *n* = 6; study 2, *n* = 8	Anodal over T3, cathodal over the contralateral Fp2	20 min	Study 1 = 1.5	35	Sham	Lower limbs	Study 1: cycling TT 20 km cycling; Study 2: cycling 25 min at 55% of peak power + TTF at 75% of peak power	No improvement
				Study 2 = 2.0					
Angius et al., 2016b	*n* = 9	Extracephalic: anodal left M1 and cathodal over ipsilateral shoulder; Cephalic: anodal left M1; and cathodal over dorsolateral right prefrontal cortex	10 min	2.0	35	Sham and control	Right knee extensors	Isometric TTF at 20% MVC	Improvement with extracephalic montage

*M1, Primary motor cortex; MVC, maximal voluntary contraction; TT, time trial; TTF, time to task failure*.

Selected studies were divided into either single joint isometric or whole body exercise. While whole-body exercise better represents real sporting competition, single-joint exercises potentially permit a better, and more controlled exploration of the physiological mechanisms associated with fatigue. This distinction is fundamental as the two exercise modalities differ in terms of metabolic, cardiorespiratory, and psychological demand, and therefore differently affect brain activity (Sidhu et al., 2013). Studies were then ordered according to publication date.

The aim of this mini-review is to provide a framework to discuss and analyse the studies involving acute administration of tDCS with the aim of improving exercise performance. A brief analysis of the physiological and psychological mechanisms and methodological limitations has been provided in order to improve the understanding of the effect of tDCS on exercise performance.

## Studies on single joint isometric exercise

The first study investigating the effect of tDCS on exercise performance was performed by Cogiamanian et al. (2007), and was comprised of two experiments. In the first, participants were divided in two groups (brain polarization and control) with both completing two elbow flexor isometric time to exhaustion (TTE) tasks. Prior to the second task, the brain polarized group received anodal or cathodal tDCS while the control group did not receive any tDCS administration. The second experiment aimed to monitor the corticospinal response following tDCS administration. No changes in MVC or EMG activity were found, but the second TTE was significantly longer following anodal tDCS, with a significant increase in corticospinal excitability observed in the second experiment. The authors were not able to provide a precise explanation for the improvement in TTE, but suggested that tDCS could act upstream of the M1 by facilitating the supraspinal drive or by protecting the M1 from inhibitory feedback arising from working muscles.

Two different studies partially replicated the study of Cogiamanian et al. (2007). Kan et al. (2013) performed a crossover study where participants performed a protocol similar to that used by Cogiamanian et al. (2007), but with a lower contraction intensity (30% MVC) and different tDCS montage (see Table 1). No changes in MVC, torque fluctuation, EMG, and perceived pain were found, with no improvement in TTE duration. The study of Muthalib et al. (2013) mainly aimed to monitor level of prefrontal oxygenation, and similarly to Kan et al. (2013), there was no improvement in MVC or TTE duration, along with no changes in prefrontal oxygenation following tDCS. However, Muthalib et al. (2013) monitored oxygenation in an area distant to the tDCS electrode location (M1), which might explain the lack of change in prefrontal oxygenation. Unfortunately, none of the above studies monitored the corticospinal response and therefore it is not possible to establish whether tDCS was able to increase corticospinal excitability.

A further experiment investigating the effect of tDCS on sustained isometric contraction was performed by Williams et al. (2013). In a crossover study, participants were asked to perform an isometric TTE at 20% MVC of the elbow flexors. Initially, no improvement in performance after anodal tDCS (compared to sham) was observed. Subsequently, the investigators divided participants in two sub groups: one group where TTE time was shorter than tDCS administration time (*n* = 8), and one group where TTE time was longer than tDCS administration time (*n* = 10). The first group showed a significant improvement in performance compared to the second. No significant changes in motor-evoked potentials (MEP) were found between conditions or group, but ratings of perceived exertion (RPE) were significantly reduced in the anodal tDCS condition. The subdivision of the participants according to task duration raises some doubts regarding the true efficacy of tDCS, and the experimental findings question whether tDCS is beneficial only when stimulation occurs during exercise and only to those with lower endurance capacity.

With the aim to provide a better understanding of tDCS mechanisms, Abdelmoula et al. (2016), monitored several muscles in a similar protocol to Cogiamanian et al. (2007). Similar to the findings of Cogiamanian et al. (2007), TTE duration was longer following anodal tDCS. However, this occurred in the absence of any change in neuromuscular, corticospinal or perceptual parameters. In fact, MVC, coefficient of variation of torque, EMG activity during exercise, MEP responses, and RPE did not differ between conditions. Because of the increase in TTE duration in the absence of changes in neuromuscular or corticospinal response, the authors proposed that the large tDCS electrode might have facilitated adjacent brain areas which affected the sensorimotor integration and the associated cognitive demand during the task without producing any change in the central motor command. This study however did not provide any evidence to support this suggestion.

The benefits of tDCS have been extended to older populations (Oki et al., 2016), with older adults being shown to have lower cortical excitability following tDCS than younger adults (Oliviero et al., 2006). Together with an increase in TTE duration after anodal tDCS, a slower increase in RPE was observed in agreement with previous experiments (Williams et al., 2013; Okano et al., 2015; Angius et al., 2016b). The authors (Oki et al., 2016) suggested that the increased excitability of the M1 could have reduced the neural drive necessary to perform the task, which therefore lowered RPE. An association between the magnitude of the effect of tDCS and baseline level of muscle strength was found (*r* = –0.55; *p* = 0.05). This may suggest that weaker subjects could receive more benefits compared to stronger subjects, although the authors did not further investigate this potential. Only 45% of the subjects demonstrated a positive response to tDCS, and so these findings might also in part explain the different outcomes across tDCS studies, as the efficacy of tDCS might rely on high responder participants. Future studies should therefore take into account such variables when determining the participant cohort.

Angius et al. (2016b) compared the effect of two tDCS montages (see Table 1) on TTE of knee extensors. TTE was significantly longer when an extracephalic montage was used without any effect on corticospinal and peripheral parameters. A reduction in RPE was found when the extracephalic montage was used, while HR and pain were unchanged. As no effect on corticospinal and peripheral parameters was found, the exact mechanisms explaining the improvement in TTE are still uncertain. However, the absence of effect on the corticospinal response could be due to the contraction intensity used (50% MVC) for the neuromuscular assessment. Indeed, the largest MEP response has been shown to occur at 50% MVC (Goodall et al., 2014), which could have masked the tDCS effect on this variable. This study suggests that an extracephalic montage is more appropriate for the improvement in exercise capacity, and could explain the null effect of tDCS shown in previous studies involving whole body exercise (Angius et al., 2015b; Barwood et al., 2016).

## Studies on whole body dynamic exercise

The first study investigating the effect of tDCS on whole body exercise was conducted by Okano et al. (2015). In a crossover, randomized experimental design, participants performed maximal cycling exercise up to volitional exhaustion. Following anodal tDCS, maximal power output improved by ~4%, and RPE and HR were lower compared to a sham condition (although they were not affected in the latter stages of the test). The authors suggested that anodal stimulation could have affected the activity of the insular cortex, thus reducing RPE and leading to an improvement in performance.

Angius et al. (2015a) investigated the effect of tDCS on exercise-induced muscle pain during cycling TTE and on pain perception during a cold pressor test. The authors did not find changes in TTE duration and physiological or perceptual parameters during exercise. However, following tDCS a significant reduction in perceived pain during the cold pressor test was found. The lack of effect during cycling was likely caused by the different type of pain stimulus, pain intensity perceived, or the attentional focus during each task. Furthermore, the authors suggested that the lack of effect on exercise performance could have been due to the tDCS montage used (Table 1), as any benefits from the anodal electrode on the M1 could have been negated by the cathodal electrode over the dorsolateral prefrontal cortex. The authors therefore suggested that a bilateral extracephalic tDCS montage would be more appropriate for whole body exercise.

An improvement in cycling TTE following tDCS was demonstrated by Vitor-Costa et al. (2015). Despite the effect on TTE, no changes in mood, physiological, or perceptual parameters were reported. It should be noted that a trend for a lower RPE following anodal tDCS was found (*p* = 0.07), suggesting that the increased M1 excitability could have made exercise feel easier for a given intensity (Williams et al., 2013; Abdelmoula et al., 2016; Angius et al., 2016a). The authors suggested that the improvement in TTE was the consequence of an increase in intracortical facilitation and M1 excitability, although this hypothesis could not be confirmed as the necessary corticospinal parameters were not monitored. In addition, the tDCS montage in this study placed one electrode over the occipital protuberance, and as a consequence the direction of current between the two electrodes could have interfered with other brain areas, thus affecting both physiological and perceptual parameters.

Angius et al. (2016a) showed an ergogenic effect of tDCS in whole-body exercise, with TTE duration increasing following anodal tDCS, paralleled a lower RPE. There were no differences observed in the cathodal and sham tDCS conditions. Following anodal tDCS, an increase in corticospinal excitability of the knee extensor muscles was also reported, leading the authors to suggest that the increased excitability of the M1 could have augmented the output to the working muscles by consequently reducing the central command required. This could have caused the lower RPE, leading participants to perceive the exercise as easier. However, no further evidence to support this hypothesis was provided, and so speculation on such a mechanism should be treated with caution.

In two separate studies, Barwood et al. (2016) investigated the effects of tDCS on a 20 km cycling time trial and a TTE test in hot conditions. The same montage used by Okano et al. (2015) was applied with the hypothesis that tDCS would reduce the RPE for a given intensity and therefore improve cycling performance. No changes in performance in either exercise protocols were found, with no differences in RPE. Unlike Okano et al. (2015) no reduction in HR following tDCS was reported. As proposed by the authors, the discrepancy in exercise outcome compared to Okano et al. (2015) might have been caused by a non-appropriate blinding procedure, and the lack of effect in HR may have been due to the high work rate adopted. The null effects may also have been due to the negative effect of the cathodal electrode. Furthermore, hyperthermia has been well-demonstrated to induce changes in metabolic and cardiovascular demand together with an increase in central fatigue (Nybo and Nielsen, 2001), which may negate any benefits of anodal stimulation.

## Possible mechanisms of actions and limitations

Collectively, experiments to date provide interesting insights regarding the possible ergogenic effects of tDCS on exercise in healthy individuals. Despite the differences across each study regarding the experimental design, task performed, and tDCS montage, there are some experimental findings which are similar across the various experiments. Firstly, acute tDCS over the M1 does not seem to improve maximal isometric force capacity (Cogiamanian et al., 2007; Kan et al., 2013; Williams et al., 2013; Angius et al., 2015b, 2016a,b). Secondly, tasks performed at a submaximal intensity are generally improved by tDCS (Cogiamanian et al., 2007; Williams et al., 2013; Angius et al., 2015b, 2016a,b; Abdelmoula et al., 2016). Thirdly, none of the physiological or neuromuscular parameters (aside from corticospinal excitability) during exercise seem to be affected by tDCS.

Regarding the inconsistency across each study, previous research has demonstrated a range of responses following tDCS stimulation from little or no effect, to a large effect with high variability in corticospinal excitability (Horvath et al., 2015, 2016; Madhavan et al., 2016). Moreover, there is an absence of a standardized and reliable protocol to monitor the effect of tDCS on the neuromuscular response (Madhavan et al., 2016). Therefore, it is not surprising that improvements in performance were accompanied with no changes in neuromuscular function with particular interest on the corticospinal pathway. Finally, the absence of rigorous blinding procedures in a considerable number of studies (see Table 1) might contribute to the mixed results currently seen in the literature, and so where this is apparent the results must be interpreted with caution.

The exact mechanisms by which tDCS improves exercise performance are still unknown. It is suggested that tDCS likely facilitates the M1 by increasing its output during exercise and *possibly* reducing supraspinal fatigue (Cogiamanian et al., 2007; Williams et al., 2013). However, this hypothesis is in contrast with previous studies as the improvement in performance appears not to rely on changes in corticospinal response (Abdelmoula et al., 2016). Other authors suggest that the lower RPE following tDCS administration might explain the improvement in performance (Okano et al., 2015; Angius et al., 2016a,b). Changes in RPE have been related to the magnitude of central motor command originating from activity of motor/premotor brain areas (de Morree et al., 2012, 2014). Thus, if M1 excitability is increased following tDCS administration, it needs to receive less input to generate the amount of output required to recruit the muscle, hence, a lower RPE for a given force or power should be expected. This hypothesis is supported by previous experiments involving non-invasive brain stimulation where manipulation of premotor and motor brain areas induced variations in RPE (Goodall et al., 2013; Takarada et al., 2014; Zénon et al., 2015). However, because of the electrode size, the effects of the tDCS could possibly influence adjacent areas by influencing the sensorimotor integration during muscular contraction without affecting the motor command (Abdelmoula et al., 2016). To the best of our knowledge no studies have monitored the activity of brain areas during exercise following tDCS stimulation and therefore development of a mechanistic understanding is a clear priority.

## Conclusion and perspectives

The promising outcomes of tDCS on exercise performance have recently attracted attention for its potential to be used domestically for ergogenic purposes. Unlike TMS equipment, tDCS devices are relatively small and easy to use and therefore its use by people unaware of its potential effects has been reported (Reardon, 2016). Given the uncertain mechanisms and the inconsistency of outcomes of tDCS prior to exercise, the use of tDCS prior to/during exercise should be treated with some caution. Future research should seek to identify the mechanisms underpinning the apparent ergogenic effect of tDCS, and focus should also be given the effects of long-term use. As tDCS is clearly of interest not only to the scientific, but also the public and commercial communities, researchers and publishers have a responsibility to disseminate transparent and objective studies that can further our understanding of tDCS.

Currently, the different outcomes observed in tDCS research are likely a consequence of differences between exercise type and/or tDCS set up (Table 1), and many of the aforementioned studies were not designed to specifically assess the mechanism by which performance was hypothesized to improve. Therefore, more studies which systematically control the tDCS variables (e.g., montage, duration, location etc.) and allow assessment of the mechanisms are required.

## Author contributions

LA was involved with the conception of the content, the writing of the manuscript, the drafting process, the revisions of the manuscript, and provided approval of the final version. He is accountable for all aspects of the work. JH was involved with the conception of the content, the drafting process, the revisions of manuscript, and provided approval of the final version. He is of the final version. He is accountable for all aspects of the work. AM was involved with the conception of the content, the writing of the manuscript, the drafting process, the revisions of the manuscript, and provided approval of the final version. He is accountable for all aspects of the work.

### Conflict of interest statement

The authors declare that the research was conducted in the absence of any commercial or financial relationships that could be construed as a potential conflict of interest. The reviewer FF and handling Editor declared their shared affiliation, and the handling Editor states that the process nevertheless met the standards of a fair and objective review.
